# Oligodendrocyte precursor cells in the substantia nigra: Implications for Parkinson’s disease

**DOI:** 10.4103/NRR.NRR-D-25-00767

**Published:** 2025-09-29

**Authors:** Julia C. Fitzgerald, Friederike Pfeiffer

**Affiliations:** Hertie Institute for Clinical Brain Research, Department of Neurodegenerative Diseases, University of Tübingen, Tübingen, Germany; Institute for Physiology, Department of Neurophysiology, University of Tübingen, Tübingen, Germany

**Current evidence for oligodendrocyte precursor cell involvement in Parkinson’s disease:** Oligodendroglial cells comprise a large cell population in the substantia nigra (SN). We recently reported a stable portion of oligodendrocyte precursor cells (OPCs) in the SN pars compacta (SNpc) in a 1:1 ratio with dopaminergic neurons: 15% of cells in neonate and young mice, rising to 20% in aged mice. Moreover, this portion represents only 45% of all oligodendroglial cells in the SNpc and 54% of all oligodendroglial cells in the SN pars reticulata (SNpr) (Fitzgerald et al., 2025). The SN is a deeply located area of the midbrain and the site of dopaminergic degeneration in Parkinson’s disease (PD). Recent advancement of single-cell transcriptomics revealed the involvement of non-neuronal cells in PD, with PD risk variants being strongly associated with oligodendroglia (reviewed by Salazar Campos et al., 2025).

The oligodendroglia (an umbrella term for OPCs, pre-myelinating and myelinating oligodendrocytes) are often highlighted in PD studies looking at genomic or transcriptomic data comparing *post mortem* brain tissue. Briefly, if we look at an overview of the role of OPCs in the midbrain specifically (for a comprehensive review see Salazar Campos et al., 2025), Martirosyan et al. (2024) highlighted association of the PD gene *LRRK2* to OPCs and Smajic et al. (2022) identified glial-specific stress signatures in PD midbrain. Dehestani et al. (2024) found overall the most significant PD GWAS risk loci in oligodendrocytes and the OPCs. Bryois et al. (2020) showed that there is a shift in oligodendroglial gene expression that occurs in PD Braak stages 1 and 2. Further work shows that transcriptional changes in OPCs may be even more important to contribute to disease development in early stages of PD compared to those in oligodendrocytes (Zhang et al., 2024).

Interestingly, the number of OPCs in the PD brain is reportedly higher than in healthy controls (Dehestani et al., 2024). OPCs exist in regulated pools keeping their capacity to proliferate and differentiate into mature oligodendrocytes throughout lifetime. The canonical view is that in the brain, OPCs migrate to axonal sites and differentiate towards mature oligodendrocytes that then differentiate and go on to wrap and myelinate neuronal projections. Dopaminergic (DA) neurons projecting from the SN are generally assumed to be scarcely or thinly myelinated, a feature that is thought to contribute to their vulnerability. But very few studies have assessed myelination of these cells in detail, with almost no reliable evidence from the human brain to back up such assumptions.

OPC-neuronal soma interactions have so far been described in the mouse cortex, hippocampus, thalamus, hypothalamus, and amygdala with both, inhibitory and excitatory neurons (Fang et al., 2025), and in the midbrain in the SNpc and ventral tegmental area with dopaminergic neurons (Fitzgerald et al., 2025) in both the developing and adult brain. The mechanisms behind these interactions remain to be discovered and can thus far only be speculated about. A recent study observed mitochondria in OPCs *versus* differentiating and myelinating oligodendrocytes in the cerebral cortex (Bame and Hill, 2024). Loss of mitochondrial quality is a key feature of PD and studies addressing cell type specific mitochondrial mechanisms are greatly needed. Bame and Hill (2024) found that OPCs contain more highly motile mitochondria than newly formed oligodendrocytes. They also found significantly higher numbers of mitochondria when compared to mature oligodendrocytes, where the mitochondria remain more stationary and shorter in shape. A study by Fang et al. (2025) focused on lysosomes, another cellular organelle with strong involvement in PD. They found that the contacts between OPCs and neuronal somata are dependent on neuronal activity and that the purpose of this interaction is to facilitate exocytosis of neuronal lysosomes. Fang et al. (2025) reported lysosome accumulation and altered metabolism in aging and Alzheimer’s disease and focused on gray matter regions, including the cortex, hippocampus, thalamus, hypothalamus, and amygdala. Since Zhang et al. (2024) reported an increase in genes involved in OPC to neuron cell-cell communication in PD, it will be interesting in the future to assess organelle morphology and cell-cell communication together with a histopathological analysis of the midbrain in PD.

**Mechanistic insight and emerging hypotheses:** This abundance of oligodendroglial cells, in particular OPCs and their contact with neuronal somata in the SN suggests additional, non-myelinating roles and raises questions about which functions they exert in both health and in PD.

One question that needs to be clarified is whether the oligodendrocytes contribute to myelination and axon health in the SN and striatum in a similar way as other gray (or white) matter regions, and which specific axons they myelinate. This is an important question in the context of PD pathogenesis because the dopaminergic striatal axons were shown to degenerate first, progressing to complete loss of the neuronal cell bodies in the SN (Gonzalez-Rodriguez et al., 2021). Considering OPC-neuronal soma interactions are most likely bidirectional, one can imagine additional roles for DA neurons to support OPCs. These functions could be mediated by the release of dopamine, which can be detected by OPCs possessing dopamine receptors (Rosin et al., 2005). Whether these contacts in the SN are supportive, regulatory, or serve structural stability is unknown, as there is very little work in this area.

Since DA neurons exhibit pacemaking activity and have large energy demands it would be interesting to know whether OPC-DA neuron interactions are needed for the provision of specific substrates, intermediates or local energy budgeting. Work on astrocyte-neuron interactions have been better described and knowing that neurons take up substrates from neighboring glial cells to meet their energetic requirements under stress conditions, it would not be surprising that OPCs, a population of cells in the brain capable of continual renewal could contribute to allostasis within the SN (in this context, allostasis is a temporal, physiological process regulating systems to anticipate energetic or biochemical needs within the network of cells in the region). Therefore, it would be interesting to investigate the possibility of mitochondrial transfer between OPCs and DA neurons. Mitochondria are not only an energy source, but they are also a source of oxidative stress and damaged mitochondria (or parts of damaged mitochondria) need to be removed from cells to maintain a healthy mitochondrial network. It is, therefore, possible that mitochondria, like the lysosomes described by Fang et al. (2025) can be exocytosed from one cell and taken up by another. The purpose could be both for removal of damaged organelles or replenishment of healthy ones.

Defining relevant biological pathways using multi-omics data will help guide functional work in tissue and induced pluripotent stem cell models. So far, pathways related to tau-protein kinase activity, regulation of inclusion body assembly, and protein processing involved in protein targeting to mitochondria are enriched in the *post mortem* brain study (Dehestani et al., 2024).

Notably, both neuroinflammation and mitochondrial dysfunction are hallmarks of both PD and myelination diseases such as multiple sclerosis. Mainstay treatments for multiple sclerosis rely on drugs that act on the immune system and there are currently no treatments that promote remyelination available. The mitochondrial (complex I) acting diabetes drug Metformin has long since been discussed for its neuroprotective properties and is currently in early clinical trials for multiple sclerosis (Al Jaf et al., 2024), since it was found to restore the capacity of aged OPCs to differentiate, thus improving the efficiency of remyelination (Neumann et al., 2019). Finally, *SNCA* mutations can cause PD. These mutations affect the production of misfolded or an increased amount of alpha-synuclein protein, which aggregates and forms a key component of Lewy body inclusions within cells, a pathological hallmark of PD. Alpha-synuclein can also aggregate in glial cells, including the mature oligodendrocytes, a feature of multiple system atrophy, an atypical Parkinsonism disorder. Alpha-synuclein delays OPC maturation by downregulating myelin basic protein (May et al. 2014). Since the accumulation of alpha-synuclein is a key pathway underlying PD and Parkinsonism, it is important that OPC function, including their proliferation and maturation, should be studied in the disease using a broad modeling approach to overcome current limitations.

**Limitations:** Studying the midbrain is challenging due to the diverse and intricate network of neurons and its deep localization, rendering subcortical *in vivo* imaging in rodent models limited to magnetic resonance imaging, positron emission tomography, and *in vivo* imaging system imaging systems. Consequently, most of the research requiring specific labeling (with antibodies for example) has been performed in tissue sections or primary cells and cell type specific tracers (dopamine transporter for example) in human imaging. A recent study carried out by Mosharov et al. (2025) mapped the diversity of a coronal section of the human brain, focusing on mitochondrial function, the first study of its kind to achieve a large amount of biochemical detail across brain cell subtypes from frozen tissue. Next steps for PD researchers would be to look more closely at cell type specificity and function in the midbrain in both health and disease.

Studying OPC-neuron interactions and *in* *vivo* processes in disease context has been complicated by the diversity of phenotypes in PD animal models, with few genetic mouse models reproducing the pathological hallmark of nigro-striatal neurodegeneration. Injection of mitochondrial toxins such as MPTP (1-methyl-4-phenyl-1,2,3,6-tetrahydropyridine) or 6-OHDA (6-hydroxydopamine), which can induce such degeneration in animals, is more invasive and less frequently used due to a combination of factors, including the 3R principle (more strict policies in licensing and the emergence of organoid modeling). Nevertheless, studying the interplay and reciprocal interactions between OPCs and DA neurons in the highly specialized SN in its natural surroundings in the midbrain including all neurotransmitters and signaling molecules present in the area, still requires the use of an intact and entire organism to clarify complex and interconnected mechanisms. We need to continue to work *in vivo*, whether vertebrates or invertebrates, as only few studies have looked at cell type specification in the midbrain comparing human induced pluripotent stem cells and *in vivo* models of PD. Since the biggest risk factor to develop PD is aging, it is also important to consider temporal interactions between OPCs and the dopaminergic neurons. Current induced pluripotent stem cell-derived cells or organoids do not fully recapitulate the aging process, but there are possibilities to address this with new technologies, for example, incorporating adult cells or material in co-cultures or rodent adult stem cells for gene editing, alongside *in vivo* and *post mortem* work.

**Figure 1:**
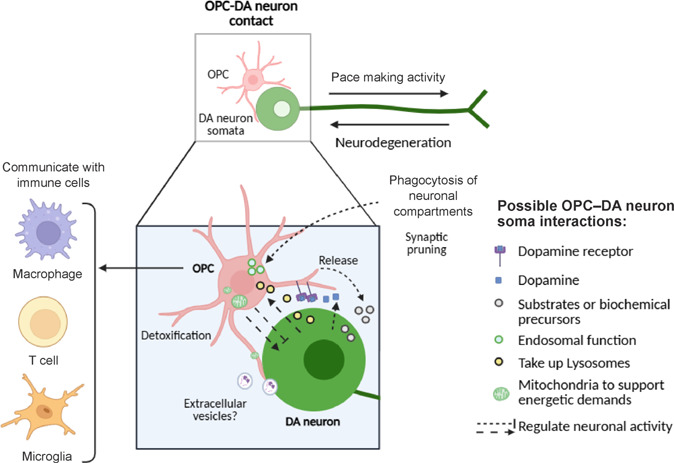
Potential functions of oligodendrocyte precursor cells (OPCs) are exerting in contact with dopaminergic (DA) neurons. OPCs contact the soma of dopaminergic neurons. Possible functions may include but are not limited to: exchange of vesicles and organelles, exchange of neurotransmitters or neurotransmitter substrates for regulating neuronal activity and/or detoxification.

**Conclusion:** The notion that oligodendroglial cells are not only abundant in the SN but also carry parts of the genetic risk to develop PD has directed much attention towards these cells. Although the role of each member of the oligodendroglial lineage in this specific area of the brain needs to be clarified, there seems to be a close and long-lasting interaction between DA neurons and OPCs in the SNpc. The mechanism behind this interaction can be uncovered by taking an interdisciplinary approach, incorporating omics data with focused studies both *in vivo* and *ex vivo*. OPCs interact with several cell types in the brain. The putative functions that may underlie these interactions are summarized in **Figure 1**. Testing hypotheses concerning the role that OPCs may play in PD and deciphering these mechanisms could provide a therapeutic entry point.
